# Pancreatic Cancer in Lynch Syndrome: A Case Report

**DOI:** 10.1089/crpc.2016.0007

**Published:** 2016-05-01

**Authors:** Madiha Gilani, Charles M. Intenzo, Voichita Bar-Ad, Harish Lavu, Ashwin R. Sama

**Affiliations:** ^1^ Division of Hematology, Department of Medical Oncology, Thomas Jefferson University Hospital, Philadelphia, Pennsylvania.; ^2^ Department of Radiology, Thomas Jefferson University Hospital, Philadelphia, Pennsylvania.; ^3^ Department of Radiation Oncology, Thomas Jefferson University Hospital, Philadelphia, Pennsylvania.; ^4^ Section Hepatopancreatobiliary Surgery, Department of Surgery, Thomas Jefferson University Hospital, Philadelphia, Pennsylvania.; ^5^ Department of Medical Oncology, Sidney Kimmel Medical College, Thomas Jefferson University, Philadelphia, Pennsylvania.

**Keywords:** pancreatic cancer, Lynch syndrome

## Abstract

**Background:** In the literature, pancreatic cancer is not frequently acknowledged among the tumors that are considered a part of Lynch Syndrome.

**Case Presentation:** Our case is one of a young man who was found, very early in life, to have pancreatic cancer. His tumor demonstrated germline microsatellite instability, and hence by definition the patient has Lynch syndrome. He responded well to treatment, which included surgery and adjuvant chemotherapy. To date he remains in remission from pancreatic cancer.

**Conclusion:** The rare instances in this case report include: (a) The patient had pancreatic cancer that fulfilled the histopathological and clinical criteria for Lynch syndrome. (b) Pancreatic cancer was diagnosed earlier in our patient than is expected in patients who suffer from pancreatic cancer as a part of Lynch syndrome. (c) Our patient had an excellent response to chemotherapy. He remains in remission to date from pancreatic cancer and is 5 years since his last treatment for this disease.

## Introduction and Background

Pancreatic ductal adenocarcinoma (PDA) is the fourth leading cause of cancer death in the United States in 2010.^1^ In 5–10% of the cases, it may be part of an inherited cancer syndrome.^2^ Lynch syndrome is an autosomal dominant disorder resulting from a germ line mutation in the mismatch repair genes MLH1, MSH2, MSH6, PMS2, and EPCAM.^3,4^ PDA associated with Lynch syndrome is less common compared with colon cancer, endometrial cancer, and ovarian cancer. Universal testing of all colon and endometrial cancers is recommended by professional societies to screen for Lynch syndrome. A strong family history of cancers associated with Lynch syndrome should prompt screening for the mismatch repair deficiency proteins by immunohistochemistry (IHC) on tumor tissue. We report a case of Lynch syndrome presenting with PDA at a very young age.

## Presentation of Case

A 29-year-old male with a history of ulcerative colitis and cerebral palsy presented to the emergency room in May 2011 with worsening periumblical abdominal pain for 4 weeks and an unintentional weight loss of 10 pounds over the prior 2 months. A CT scan of the abdomen and pelvis with contrast revealed a 5.8 × 5 cm mass within the body and tail of the pancreas, obliterating the splenic vein, and encasing the splenic artery. There was no evidence of metastatic disease on cross-sectional imaging. The patients' cancer antigen (Ca)19-9 level was elevated at 263. An endoscopic ultrasound-guided fine needle aspirate of the pancreatic mass was performed, which revealed PDA. The patient was taken for operative exploration and underwent a distal pancreatectomy and splenectomy. Pathological analysis revealed a 7.0 cm moderately differentiated adenocarcinoma of the pancreas, the presence of lymphovascular invasion. The surgical margins of resection were negative and none of the 17 resected lymph nodes harbored malignant disease. Based on this information, the patient was determined to have a stage IIA adenocarcinoma of the pancreas.

The patient had an interesting family history. Ovarian cancer and uterine cancer were diagnosed in his mother at the age of 37. Colon cancer was diagnosed in his maternal grandmother at the age of 39, and multiple basal cell and squamous cell cancers of the skin, lymphoma, and renal cell carcinoma during her lifetime. Ovarian cancer was diagnosed in his maternal aunt at the age of 37 and uterine cancer. This family history was suspicious for Lynch syndrome, and prompted us to test the tumor sample by IHC for the mismatch repair gene mutation. The test revealed a loss of MSH2, MSH6, and PMS2 by IHC and PCR showed microsatellite instability. Germline testing showed a mutation in MSH2, confirming Lynch syndrome.

**FIG. 1.**
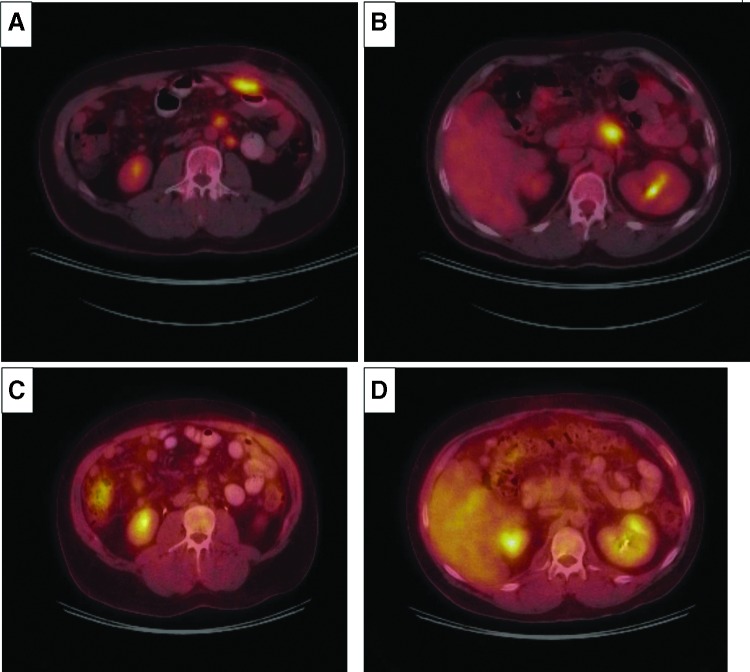
**(A, B)** Show the PET/CT on September 2011 at the time of disease relapse. **(A)** Hypermetabolic omental metastasis and hypermetabolic left para aortic lymphadenopathy. **(B)** Hypermetabilic activity within the surgical bed representing tumor recurrence. **(C, D)** Show the PET/CT on March 2012 demonstrating interval resolution of hypermetabolic activity in the surgical bed, omental metastasis, and left para aortic lymph nodes.

**FIG. 2.**
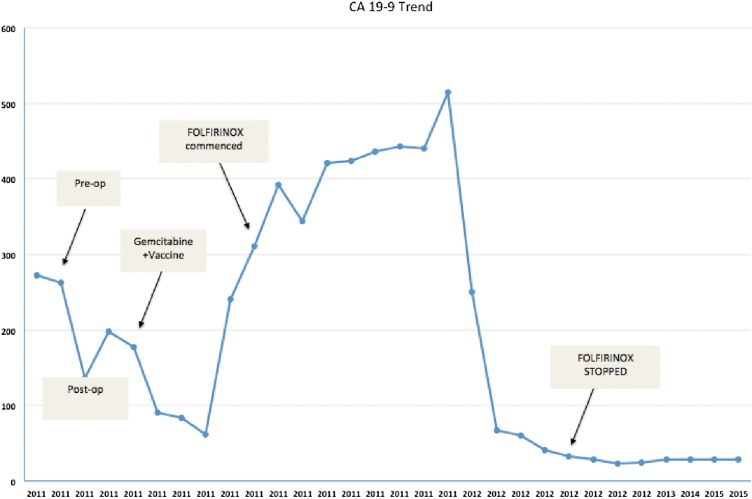
Showing cancer antigen (Ca)19-9 trend over the years during treatment of the disease.

The patient recovered well after the surgery and proceeded with adjuvant chemotherapy. He was enrolled onto a phase 3 randomized hyperacute immunotherapy study (NLG 0405) that evaluated algenpantucel-L with gemcitabine chemotherapy and was randomized to the gemcitabine alone arm. The patient tolerated gemcitabine treatment well, but unfortunately developed symptoms of abdominal pain during treatment, which led to an emergency room visit. His Ca19-9 had been trending up again (from 62 postoperatively to 424 by October 2011). An MRI was performed to further investigate the abdominal pain, which revealed a 2.2 × 2.3 cm mass in the tumor bed, with retroperitoneal lymphadenopathy. PET scan showed hypermetabolic activity (SUV max 12) in the surgical bed, omentum, pre aortic, and para aortic lymph nodes. Given these findings, his therapy was switched to a combination of folinic acid, fluorouracil, iriniotecan, and oxaliplatin (FOLFIRINOX). The patient tolerated the therapy well, and at the end of eight cycles of treatment, he experienced a biochemical and radiographic complete response per Figure 1. He received a further four cycles of chemotherapy after which his treatment was stopped. Figure 2 shows the trend of the patient's Ca19-9 level during the course of his illness. To date, our patient remains in a complete remission. He is seen for a follow-up every 3–6 months. His last visit with us was in January 2016, almost 5 years after the original diagnosis. He remains in complete remission and free of any cancer at this point. His latest Ca19-9 is normal at 28 and his last CT scan of the abdomen with contrast in January 2016 showed no evidence of recurrent or metastatic disease.

## Discussion

The overall 5 year survival of patients with PDA is estimated to be 6%.^5^ Stage II PDA has a 5 year survival rate of ∼27%.^5^ Our case demonstrates a rare scenario, in which a patient with Lynch syndrome was found to have PDA at a young age. Most patients who have developed PDA in the setting of Lynch syndrome have been diagnosed after the age of 40. Kastrinos et al.^2^ conducted a study with an objective to estimate PDA risk in 147 families who had germ line mismatch repair gene mutations. They showed that these patients were at a nine-fold increased risk of developing pancreatic cancer as compared with the general population. This case strengthens the idea that PDA is indeed a part of the list of extra colonic cancers that are seen in Lynch syndrome. A detailed family history may be suggestive of this syndrome and should prompt testing for the mismatch repair gene defect. Positive testing should prompt the involvement of a genetic counselor to manage these patients appropriately. We suspect that this patient had a recurrence of PDA during adjuvant therapy, but when the treatment regimen was altered to FOLFIRINOX, he remarkably experienced a complete response. Today, the patient remains with no evidence of disease nearly 5 years after pancreatic cancer was diagnosed.

## Abbreviations Used

Ca: cancer antigen
CT: computed tomography
IHC: immunohistochemistry
MRI: magnetic resonance imaging
PCR: polymerase chain reaction
PDA: pancreatic ductal adenocarcinoma
PET: positron emission tomography
